# Supplementary material for: Are carbon emissions trading and green financial instruments synergistic? -Comprehensive quantitative research based on content analysis
Source: PLoS One. 2024 Mar 7;19(3):e0298601. doi: 10.1371/journal.pone.0298601 (PMC10919601; doi:10.1371/journal.pone.0298601)
Supplement: S1 Data — (ZIP) [file pone.0298601.s001.zip › Data/地级市Z_F工作报告.zip!/╜¡╦╒/╗┤░▓╩╨/└┤╫╘╣1⁄2╓┌║┼í╛┬φ┐╦╩2╛▌═°í┐_2019.docx]

各位代表：

现在，我代表市人民政府向大会作工作报告，请予审议，并请各位政协委员和其他列席同志提出意见。

一、2018年工作回顾

过去一年，面对严峻复杂的宏观环境、艰巨繁重的发展任务，我们坚持以习近平新时代中国特色社会主义思想为指引，全面贯彻党的十九大精神，按照党中央、国务院、省委省政府和市委的决策部署，牢牢把握稳中求进总基调，深入践行新发展理念，以供给侧结构性改革为主线，统筹抓好改革发展稳定各项工作，经济社会保持平稳健康发展，较好完成了各项目标任务，高质量发展实现良好开局。

（一）坚持量质并举，经济运行稳中有进。实现地区生产总值3601亿元、同比增长6.5%，规模以上固定资产投资增长9%，一般公共预算收入247.3亿元、增长7.2%，城乡居民人均可支配收入为35828元、17058元，分别增长8.6%和9.3%。突出战略引领。由我市首提首推的淮河生态经济带上升为国家战略，填补了淮安长期没有国家战略直接覆盖的空白，积极参与编制大运河文化保护传承利用规划纲要、江淮生态经济区规划，更多淮安元素融入国家、省重大战略。抓紧用好省委省政府支持淮安建设航空货运机场的战略机遇，系统谋划推进向上争取、对外招引、规划编制等前期工作。筑牢产业支撑。重点打造新一代信息技术、新能源汽车及零部件、盐化凹土新材料、食品等优势特色产业，工业经济稳健运行，规上工业投资、产值、应税开票均保持两位数增长。现代物流、现代商贸、生态文旅等特色服务业加快发展，服务业增加值增长8.8%，居全省前列。淮安电子商务现代物流园创成省级示范物流园区，盱眙成为省乡村旅游创新发展示范区，龙宫大白鲸世界、金湖水上森林获批国家4A级景区，创成一批省5星和4星级乡村旅游区。特优高效种植、特种绿色水产、特色生态休闲等高附加值高颜值农业产业提速增效，发布实施“淮安大米”系列标准，新增高效设施园艺面积6.8万亩，稻虾综合种养规模突破35万亩。建成涟水万亩“百药园”，白马湖赏花游等休闲农业持续升温，4个县区跻身“全省粮食生产全程机械化整体推进试点县”。坚持项目带动。220个市级以上重大项目完成投资1342亿元，新增投资超亿元内资项目210个，其中超10亿元项目30个，新开工亿元以上工业项目154个，新竣工设备投资超千万元项目107个。德淮一期、时代芯存等龙头项目主体竣工，首辆“淮安造”纯电动新能源汽车正式亮相。实施投资超5000万元服务业项目200个，华强方特、中国漕运城等重大项目开工建设。新竣工30个投资超亿元农业项目，新增9户省级农业龙头企业。完成水利重点工程投入30亿元，淮河入江水道整治等三大流域性治淮骨干工程全面收官。强化创新驱动。高新技术产业产值增长21.4%，获批135户国家高新技术企业，创历史新高。组建智能装备制造、盐化新材料产业联盟，新增市级以上“两站三中心”66个、省两化融合贯标试点企业26户，实现国家级工程研究中心、省级双创示范基地零的突破。建成市技术产权交易市场，新增中国驰名商标2件。引进培育“淮上英才计划”创新创业领军人才68人、团队11个，专利授权量、万人有效发明专利拥有量分别增长66.9%和12.5%，创新成为推动高质量跨越发展的重要动力。

（二）坚持攻坚突破，改革开放蹄疾步稳。围绕让“盆景”变“风景”，重抓各项举措落地生效。重点领域改革实现突破。加大放管服改革力度，全面实施三十证合一和证照分离改革，常态化运行“35745”审批服务新流程，积极推广模拟审批、代办制等创新做法，开展减证便民专项行动，健全“双随机、一公开”监管机制，全面启动市县镇村一体化审批服务体系改革试点，“数字化联合审图”获国务院第五次大督查通报表扬，“不见面审批”列入省标准化试点。扎实推进新型城镇化和土地使用制度改革，全面实施乡镇布局优化，原定两年任务一年完成，有序开展高沟经济发达镇行政管理体制改革。五大功能片区土地利用规划顺利实施，洪泽区完成“双减量”试点万亩整治目标。财税金融改革成效明显，顺利完成国税地税征管体制改革，组建市融资担保集团，中信银行淮安分行开业，金融机构本外币贷款增幅全省第三，政府性债务和金融风险防控机制进一步健全。农村改革深入推进，全面开展农村集体资产清产核资，推广联耕联种、农地入股等经营模式，新增省级示范家庭农场23个、合作社51个。农业水价综合改革成效显著。对外开放实现突破。新设外资项目168个，其中超亿美元项目16个，制造业利用外资占比40%。积极应对中美经贸摩擦和国际贸易新形势，落实投资贸易便利化各项措施，培育外贸新增长点企业150户，淮安新港正式开通外贸航线，外贸进出口增长8.1%。新增国际友好交流城市2对。台资高地建设实现突破。召开台资集聚示范区首次省联席会议，编制相关发展规划，深入开展淮台经贸、文化、医疗等合作交流，强化淮台产业融合发展。举办台湾·淮安周、台商论坛·2018台湾周等活动，落实淮安昆山台资经济协同发展机制，新引进及增资台资项目48个，其中超千万美元项目增长45%。深化101%服务品牌建设，开展“与台企同行”系列活动，连续7年获评台商投资“极力推荐城市”，在参评112个大陆城市中排名第13位，比上年度提升6位。平台载体建设实现突破。7家省级以上开发区在全省排名全面进位，淮安经济技术开发区上升至第12位。苏淮高新区成为省“263”专项行动启动后唯一通过扩区环评的化工园区，清河开发区获批省级经济开发区，宁淮现代服务业集聚区建成双创科技园等项目，5家南北共建园区获省通报奖励，各类园区承载力集聚力不断增强。

（三）坚持融合发展，城乡建设协调推进。着力优化布局、完善功能、提升品质，打造宜业宜居环境。中心城市建设扎实推进。突出规划引领，加强城市总体设计，投入961亿元、实施725个城建重点项目。加快里运河文化长廊、高铁商务区建设，开工淮安东站综合客运枢纽等重大项目，竣工金融中心一期工程，计划入驻金融机构等企业80户。市区内环高架一期及连淮扬镇、徐宿淮盐铁路进展顺利，淮安机场二期扩建工程投入使用，金湖通用机场开工建设。坚持精准施策，房地产市场保持平稳，住房公积金管理模式不断创新。改造棚户区132万平方米，整治提升老旧小区180个，新增停车泊位1230个，完成淮海地下广场提升改造。新设永久性绿地26块，新增改造绿地217公顷，城镇绿色建筑占比75%，7户企业获批省建筑产业现代化基地。全面启动新一轮全国文明城市创建，发布淮安市民文明行为“十不准”，强化“门前五包”责任制，高分通过国家卫生城市复审。镇村建设全面提速。大力实施乡村振兴战略，制定农民群众住房条件改善五年行动计划，出台集体土地新建农民集中居住区项目审批和招投标指导意见，编制农民群众居住空间布局规划，完成市级特色田园乡村设计方案，新开工农民集中居住区29个、建成4个，搬迁村居111个、2.2万户。施河智教乐享小镇、淮安高新区智芯小镇、蒋坝河工风情小镇入选第二批省级特色小镇创建名单，三河镇创成国家卫生镇。建成农村公路567公里、桥梁152座，行政村四级公路覆盖率超过97%，镇村公交实现全覆盖，提前完成农村饮水安全巩固提升任务。启动乡镇垃圾分类试点，建制镇污水集中处理率50.6%。生态文明建设持续发力。获批全国第一批生态文明先行示范区试点城市，通过国家级水生态文明城市建设验收，盱眙、洪泽入选首批省级生态文明示范县区。深入开展“263”专项行动，全面整改落实中央环保专项督察“回头看”和省级环保督察反馈意见与交办问题。实施382个大气污染防治重点项目，空气质量优良天数254天、占比提高1.2个百分点，市区PM2.5平均浓度与上年持平。全面排查治理化工企业遗留地块，新增固废处理能力3.75万吨。积极实施化工企业“四个一批”专项行动，加快大运河沿岸化工企业搬迁，关停化工企业30户。创新河、湖、断面“三长一体”管护机制，新建生态样本河道62条，整治城市黑臭水体17条，入选全国20个黑臭水体治理示范创建城市，竣工南水北调东线一期淮安段截污导流工程，国考断面水质和县级以上集中式饮用水源地水质全部达标。生态环境满意率居全省第三，生态正成为淮安最靓丽的底色。

（四）坚持以人为本，民生保障持续增强。不断加大民生投入，完成十件民生实事项目，着力提升群众的获得感、幸福感、安全感。脱贫攻坚成效显著。落实“阳光扶贫”监管机制，深入开展人大代表“千百万扶贫济困大行动”等结对帮扶活动，加快推进古淮河沿线、淮涟结合部和西南岗片区等扶贫产业带建设，实施产业富民项目114个，落实社会救助和兜底保障政策，12万低收入人口如期脱贫。通过开展农村相对集中居住、土地连片整治、宅基地有偿退出试点和资产收益扶贫等工作，51个省、市定经济薄弱村达标出列。公共服务持续提升。新建中小学校、幼儿园53所，全省中职技能大赛获历史最好成绩，创成省现代化专业群和实训基地12个，督促190家校外培训机构整改达标，教育现代化监测、省对设区市政府履行教育职责考评得分苏北第一。公立医院综合改革获国务院表彰激励，分级诊疗、家庭医生签约服务制度不断完善，苏北临床医学研究院挂牌运行，与沪宁等地建立跨区域医联体12个、专科联盟10个。建成大运河淮安段文化遗产数据库，举办大运河文化带城市非遗展，依托洪泽湖国际大闸蟹节与央视合作举办首届中国农民丰收节特别节目，创作大型现代京剧《鹤舞云天》等一批文艺精品。新建体育公园32个、健身步道86.5公里，承办一批国际国内体育赛事，举办淮河生态经济带体育产业博览会。保障能力不断加强。开展就业创业技能培训5.5万人，新增城镇就业9.2万人，扶持5.6万人实现再就业。城乡低保标准分别提高到580元和520元，农村低保标准超过省定脱贫线。继续提高残疾人帮扶补贴标准，为残疾儿童提供精准康复服务。为9.7万困难老人购买养老服务，建成医养结合型养老机构30个。从快彻查严处金湖过期疫苗事件，深入开展疫苗及食品药品安全隐患大排查、大整治专项行动。食品安全逢十（食）行动获国务院专题推介，放心消费创建满意度居全省前列。扎实开展殡葬领域突出问题专项整治。社会治理有力有序。推进公共法律服务体系建设，“七五”普法通过省中期考核。推行网格化社会治理，强化人民调解、行政调解，矛盾纠纷多元化解经验成为全国8个典型案例之一，重点信访积案化解率大幅提升。推动改革强警、科技兴警，立体化现代化社会治安防控体系成效突出，各类违法犯罪案件不断下降，群众安全感全省唯一实现3年稳增长，连续2年获评省社会治安综合治理先进市。严格落实安全生产责任制，各类事故起数和死亡人数实现双下降。深化军民融合发展，顺利通过省双拥模范城验收，国防和后备力量建设稳步发展。全面推进第四次全国经济普查。国家安全、民族宗教、机关事务管理、供销、物价、气象、仲裁、档案、广播电视等工作进一步加强，老龄、青少年、妇女儿童、慈善、红十字等各项事业取得了新进步。

（五）坚持担当作为，履职能力不断提升。按照进入新时代要有新气象、新作为的要求，大力弘扬周恩来精神，切实扛起全面从严治党责任，进一步增强政府的执行力和公信力。更加注重系统谋划与协调推进。聚焦落实高质量跨越发展，扎实开展解放思想大讨论和“立标杆、补短板、学赶超”调研活动，研究出台“1+8”高质量发展方案，精心谋划构建“433”现代产业体系、实施乡村振兴战略、打造航空货运枢纽等重大引领性工作，完善统计监测和目标考核体系，着力强化与高质量发展相适应的基础支撑。全力以赴抓项目、优环境、补短板、增后劲，出台实施“惠台58条”、金融支持实体经济、深化科技体制机制改革等政策意见，设立重点产业发展基金，确保经济运行在合理区间。更加注重特色塑造与品牌引领。围绕实施“一区两带”战略，确立生态文旅水城发展定位，促进发展方式转变、结构优化和动力转换。全力打响“水懂我心、自然淮安”全域生态文化旅游品牌和“淮味千年”农产品区域公用品牌，成功举办首届中国（淮安）国际食品博览会，荣获中国食品名城称号，启动申报联合国科教文组织创意城市网络“美食之都”，进一步提升城市美誉度和影响力。更加注重依法行政与高效服务。自觉接受人大法律监督、工作监督和政协民主监督，全年办理人大代表建议200件、政协提案457件，满意率均在99%以上。严格执行重大行政决策程序，推进行政执法和行政复议应诉规范化建设，加大政务公开和新闻发布力度，法治政府建设取得新成效。认真执行中央八项规定及实施细则，严格落实党风廉政建设责任制，坚持不懈整治“四风”，切实抓好中央和省委巡视整改落实工作。加大审计监督、财政检查监督力度，创新政府投资工程建设项目标后监督等工作机制，进一步筑牢反腐倡廉防线。坚持领导干部领办重大项目、服务重点企业，狠抓“两为”专项整治，推动落实鼓励激励、容错纠错、能上能下“三项机制”，行政服务效能进一步提升。

各位代表，过去一年的成绩来之不易、令人振奋，这是省委省政府和市委正确领导的结果，是市人大、市政协和社会各界监督支持的结果，是全市人民团结奋斗的结果。我谨代表市人民政府，向广大干部群众，向人大代表、政协委员和离退休老同志，向各民主党派、工商联、无党派人士和各人民团体，向驻淮部队、武警官兵和部省属驻淮单位，向来淮投资创业者，向所有关心支持周总理家乡建设的淮安乡贤和海内外朋友，表示衷心的感谢并致以崇高的敬意！

同时我们也清醒地看到，对照高质量发展要求，经济社会发展的不平衡不充分矛盾较为突出，产业层次不高，科技创新能力不强，实体经济面临成本上升、融资结构性矛盾突出等问题，民生保障、公共服务等领域还存在诸多短板，打好“三大攻坚战”任务仍很艰巨，政府职能转变力度还要加大，少数干部担当精神和服务意识还不强。面对前进中遇到的困难和问题，我们将矢志不渝地解放思想、深化改革、扩大开放，在推进高质量跨越发展中认真加以解决。

二、2019年工作任务

今年是新中国成立70周年，是决胜高水平全面建成小康社会的关键之年。新的一年，我们面临的发展机遇前所未有，淮河生态经济带发展规划启动实施，“一区两带”建设与长三角一体化等重大战略实现优势叠加，省委省政府大力支持航空货运枢纽建设，徐宿淮盐铁路建成通车，淮安在区域发展中的地位作用进一步凸显，需要我们以更足准备抢抓机遇、乘势而上。我们面对的复杂形势前所未有，世界经济走势错综复杂、变数增加，贸易摩擦影响持续显现，国内经济下行压力加大，企业运行困难增多，区域竞争日趋激烈，重大风险挑战加剧，需要我们以更大定力勇于担当、敢于斗争。我们面前的艰巨任务前所未有，进入新时代，我们正处在转型升级的攻坚期，面临着转变观念与提升能力的双重考验，担负着提高发展质量与保持规模速度的双重任务，承载着发展经济与保护环境的双重压力，肩负着抢抓机遇与防范风险的双重责任，需要我们以更强意志不忘初心、久久为功。

新的一年，我们将以习近平新时代中国特色社会主义思想为指导，深入贯彻党的十九大和十九届二中、三中全会精神，认真落实中央经济工作会议、省委十三届五次全会和市第七次党代会第三次会议的决策部署，积极践行新发展理念，按照“变中行稳、稳中求进、进中向好、好中争前”的工作基调，聚焦“6+1”部署，以供给侧结构性改革为主线，不断深化市场化改革、扩大高水平开放，加快构建现代经济体系，全力推动高质量跨越发展，努力在全省考核中实现进位争先。今年全市经济社会发展主要预期目标是：地区生产总值增长6.5%左右，规模以上工业增加值同口径增长5.5%左右，规模以上固定资产投资同口径增长7.5%左右，一般公共预算收入增长5%，社会消费品零售总额增长8.5%，研发投入占地区生产总值比重1.91%，城乡居民人均可支配收入增长8%左右，居民消费价格指数控制在3%左右，常住人口城镇化率62.4%，城镇登记失业率控制在4%以内，节能减排、空气和地表水质量完成省定任务，以优异的成绩迎接新中国成立70周年。我们将重点办好十件要事：一是建成淮安高铁东站，开启高铁时代；二是建成市区内环高架一期，构建城市快速交通体系；三是建立淮河生态经济带城市协作机制，加快规划实施步伐；四是改善11.8万户农村群众住房条件，促进城乡融合发展；五是提前一年基本完成脱贫攻坚任务，省级重点帮扶县区全部达到“摘帽”标准；六是全面完成城区黑臭水体整治任务，还城市绿水长流；七是开通全货机航线，加快推进航空货运枢纽建设；八是实施重特大项目攻坚突破，力争新培育1户销售超百亿工业企业；九是健全市县镇村一体化审批服务体系，进一步优化营商环境；十是申创“美食之都”，推动舌尖上的一二三产业融合发展。围绕全年目标，着重抓好七个方面工作：

（一）立足更高起点，推动改革开放再出发。认真落实中央庆祝改革开放40周年大会精神，按照“九个必须坚持”的要求，奋力谱写好新时代淮安春天的故事。

纵深推进放管服改革。坚持问题导向、需求导向，建好政务服务“后台”，支撑好经济社会发展“前台”。全面升级市政务服务“一张网”，强化全市域网上办、集成办和帮办代办，实现基层群众办事“就近办”、最多跑一次。放大“数字化联合审图”品牌优势，打造“35745”升级版，推进“不见面审批”标准化，提升“互联网+政务服务”水平，真正做到审批不见面、服务看得见。全面落实减税降费政策，加强服务民营企业审计监督，进一步清理拖欠民营企业账款，切实帮助企业强信心、渡难关、稳预期，努力打造一流营商环境。

全面深化重点领域改革。推进新型城镇化综合配套改革，构建乡镇“1+4”基层管理体制，推动施河镇等集成改革试点，打造全市综合改革试验区。完成市级交通运输、生态环境、市场监管等领域综合行政执法体制改革。深化土地使用制度改革，加快五大功能片区规划实施，深入开展同一乡镇范围村庄建设用地布局调整和工矿废弃地复垦利用试点，支持盱眙、洪泽开展较大规模矿山复垦利用和“双减量”试点。加大财税金融改革力度，有效控制和化解政府性债务，严守不发生系统性区域性金融风险的底线；全面落实金融支持实体经济政策措施，推进线上综合金融服务平台和线下金融服务中心建设，打造优质高效的金融服务体系，不断优化金融信贷结构；健全财政资金支持重大项目建设等激励措施，落实市县区信用贷款风险共担机制，稳步推进平台公司转型发展。按照中央和省总体部署，全面完成政府机构改革任务，稳妥推进国有企业办社会职能剥离工作。

大力提升开放发展水平。深入开展重特大项目攻坚年活动，坚持软件与硬件并举、对外招引与对内挖潜并举、主攻二产与兼顾一三产并举、产业项目与基础设施并举、跨区域产业专业招商与兼职招商并举，重抓营商环境优化、重特大产业项目招商、重要基础设施建设、重点载体功能提升和保障激励机制创新突破，在全市营造全力招项目、建项目和服务项目的浓烈氛围。聚焦优势特色产业，新引进亿元以上内资项目200个、3000万美元以上外资制造业项目30个，制造业、战略性新兴产业利用外资占比分别达40%和30%以上，力争在百亿元项目招引上取得更大突破。放大“惠台”政策效应，用好台资集聚示范区省联席会议机制，全面推进台资集聚示范区“二次创业”。坚持优进优出，实施工业企业国际化经营行动计划，引导科技型、内销型企业加强出口，培育外贸新增长点企业100户以上。参与“一带一路”交汇点建设，支持重点企业跨境并购，对沿线国家出口占比25%以上。完成综保区二期封关验收，申请建设国际快件监管中心，创新发展跨境电商等新兴业态。以淮安经济技术开发区为龙头，推动园区创新发展，坚持“以亩均论英雄”，科学编制产业发展规划，重点培育一批技术研发、成果转化和检验检测平台，着力打造特色产业集群。

加快推进区域协调发展。发挥首提城市牵头引领作用，围绕淮安“一中心五枢纽”定位，制订落实淮河生态经济带发展规划三年行动计划，建立健全城市协作机制，积极推动组建相关领域专委会及产业联盟，共同谋划推进沿淮流域间基础设施、生态环保、产业升级、公共服务等共建共享，扎实开展淮河干流二级航道建设和沂淮、蚌淮铁路等前期工作，全力推动淮河生态经济带发展规划高标准实施、城市协作机制高效率运转。主动融入长三角一体化发展，加快实施宁淮挂钩深度合作规划，争取宁淮现代服务业集聚区创建省南北共建园区，持续做好援疆援藏各项工作，进一步加强与铁岭市的交流合作。

（二）突出扶优培强，大力发展先进制造业。坚持以创新转型为引领，加快形成龙头企业领跑、骨干企业支撑、中小企业跃进的梯次发展格局，促进制造业加快向中高端发展。

提升发展优势产业。加速产业集聚，落实“三新一特”主导产业发展行动计划，统筹推进高端装备制造、生物技术及新医药等其他战略性新兴产业，稳步发展纺织、建材等传统产业，全市规上工业投资增长9%左右，新开工亿元以上工业项目150个，新竣工设备投资超千万元项目100个。提升产业业态，继续开展“十百千”行动，加快开发高精尖产品、塑造高端品牌、培育高新技术企业，促进现有产业链条向两端延伸。引进实施光芯片、半导体层膜等关联项目，大力培育集成电路、应用电子等先进制造业集群。推动绿色发展和智能化发展，实施30个绿色改造项目，积极创建绿色工厂；扎实开展化工企业“四个一批”专项行动，全面完成“两减”年度任务；新增“上云”企业30户，3户企业通过国家两化融合管理体系贯标。

扶持壮大骨干企业。放大产业基金、百亿企业培育政策叠加效应，聚力培育规模10亿元以上、具备产业链整合能力的龙头企业，助推庆鼎精密电子开票销售突破百亿元，敏安汽车、德淮半导体、时代芯存等项目全面投产。以158户骨干企业为重点，深入推进市县镇三级培育计划，力争应税销售亿元以上企业310户、10亿元以上企业32户，新培育规模企业100户，新增市级以上科技小巨人企业、专精特新中小企业各10户以上。助推150户小微企业入规升级，确保新增规上企业60户，逐步提升私营企业、个体工商户存活率和活跃度。

增强创新驱动能力。发挥企业主体作用，实施重大成果转化项目6个以上，新获批国家高新技术企业100户以上，高新技术产业产值占规上工业总产值比重22.5%，科技进步贡献率提升1.5个百分点。发挥平台主阵地作用，推动淮安高新区、苏淮高新区争先进位，提升6个国家级“星创天地”科技内涵，新建众创空间和科技企业孵化器10个以上。发挥产学研协同创新作用，加快产业技术协同创新联盟提标扩面，力争服务企业超千户，开发和推广应用新技术新产品50项以上。发挥政策引导作用，开展知识产权融资试点，建立科技融资奖补制度，完善大型科研仪器设施等共享服务平台建设。发挥人才引领作用，落实省“双创计划”、市“淮上英才计划”，推进新型技能大军培育工程，不断夯实创新发展的人才基础。

（三）聚力提速增效，全面做优现代服务业。围绕厚植优势、提升业态、升级消费，积极打造区域引领的现代商贸中心、物流枢纽和文旅高地。

加快发展枢纽经济。立足战略、区位、产业等综合优势，以航空、高铁、内河航运三大枢纽经济区为支撑，加快规划建设综合交通枢纽，优化配置人流、物流、资金流，提升要素集聚能力。抓住航空货运枢纽列入淮河生态经济带发展规划机遇，加紧编制枢纽经济、集疏运体系等规划，力争尽早纳入国家物流枢纽布局；积极争取上级政策支持，推动与龙头企业合作共建，加快机场三期扩建前期工作。紧扣高铁建成通车节点，发展东站周边商务商贸、旅游集散等业态，完善生产生活服务功能，集聚高铁商务区人气商气。围绕打造内河航运中心目标，启动建设淮河入海水道先行段工程，全面推进新港配套项目建设，发展集散分拨、仓储配送等高附加值产业，推动内河集装箱运输量再创新高。

打响全域旅游品牌。实施全域旅游发展三年行动计划，推进国家旅游业改革创新先行区建设，进一步唱响“水懂我心、自然淮安”品牌。打造精品旅游新龙头，加快建设华强方特、中国漕运城等大型项目，推动西游记文化体验园创建国家5A级景区，创建一批国家和省级旅游度假区。塑造乡村旅游新特色，大力发展“两圈两带”休闲农业，重点推进16个特色田园乡村建设，创成一批省级乡村旅游区。创新产业旅游新燃点，加快发展“千年淮盐”等工业旅游、白马湖养生养老等休闲旅游、金湖三河湾等水利风景旅游。提升服务旅游新体验，举办淮扬菜寻味之旅、中国大运河文化旅游节等活动，加快淮安旅游吉祥物设计研发，建设智慧旅游信息平台，实现“一网在手、游遍淮安”。

集聚发展现代商贸。大力培育新兴业态，拓展宁淮现代服务业集聚区、淮安电子商务现代物流园等平台功能，带动电商产业集聚集群发展，加快实体商业和老旧市场转型升级，启动建设中国农批·淮安农产品物流园项目，实现线上线下有机融合。完善新建小区社区便民服务业态配置，加快建设15分钟便民生活圈。提升餐饮等传统服务业，弘扬淮扬美食文化，开展淮扬菜品牌店创建评选，推进老字号传承发展。大力发展会展经济，建成食品博览城，精心筹办第二届中国（淮安）食博会，进一步打造食品行业国际展会品牌。

（四）着力扬长补短，开创乡村振兴新局面。编制乡村振兴战略实施规划，推进现代农业提质增效、乡村建设集聚提升等十项行动，努力打造新时代乡村振兴的淮安样本。

大力发展“双高”农业。推进重要基地建设，聚焦“三特”产业发展壮大，整合涉农项目资金，加快集中连片系统治理，建成一批高效种养区和优势发展区，新增稻虾综合种养面积40万亩，新建高标准农田30万亩，争取涉农县区全部纳入省级粮食生产全程机械化创建试点。支持淮安国家农业科技园创建国家农业高新技术产业示范区，新增1个省级现代农业产业示范园。加快重点品牌建设，各县区分别培育1-2个重点品牌。完善农产品标准化生产和质量监管追溯体系，不断提升品牌价值，力争将“淮味千年”创成国家级农产品区域公用品牌。加大重大项目突破，实施投资超3000万元农业产业化项目100个，新增销售超亿元企业10户以上。推进洪泽湖周边滞洪区建设，建成洪泽湖流域气象灾害监测预警中心。

加快建设美丽乡村。抓住用好政策“窗口期”，全面推进农民群众住房条件改善工作，新建集中居住区62个、定向安置房5.8万套，货币安置3.3万户，改造提升特色自然村58个、惠及2.7万户。全面落实农村人居环境整治三年行动方案，加快基础设施和公共服务设施向新型农村社区延伸，新建无害化卫生户厕2万座，行政村四级公路覆盖率达98%，美丽宜居乡村建设达标率突破70%。按照每个县区“做精一个、拓展一个”的要求，着力打造12个特色小镇，引领城乡融合发展。

充分激发农业农村活力。深入推进农村土地“三权分置”和农村集体产权制度改革，建立农村集体产权市场交易机制，有序推进土地经营权抵押融资试点，稳步扩大股份合作、联耕联种等经营规模，新增土地流转面积20万亩，50%以上的村基本完成农村社区股份合作制改革。提升新型农业经营主体发展质态，新培育市级以上示范家庭农场、合作社各100个。深化农业综合行政执法改革和镇村联审工作，农业水价综合改革覆盖面80%以上，建设“三体两强”示范基层供销社10个。

全面完成脱贫攻坚目标。完善“阳光扶贫”监管系统，强化专项扶贫审计，压紧夯实各级帮扶责任，增强到村到户帮扶精准度，提前一年基本实现“建档立卡低收入人口全部脱贫，省、市定经济薄弱村全面出列，重点片区面貌显著改善”三大目标。强化重点扶贫片区、经济薄弱村项目帮扶，促进村集体和低收入农户双增收。优先安排建档立卡低收入农户住房条件改善，对重点低收入群体强化社会救助和兜底保障脱贫。高度重视返贫问题，加大临时救助力度，持续巩固脱贫攻坚成果。

（五）注重综合施策，打好污染防治攻坚战。以争创国家生态文明建设示范区为主线，加快环境治理能力现代化，努力还老百姓蓝天白云、繁星闪烁。

重拳整治环境污染。深入推进“263”专项行动，扎实开展环保督察专项整改，严格落实强制性污染减排细化实施方案，着力解决突出环境问题，推动生态环境质量走在全省前列。强化大气污染系统治理，坚持科学施策、分类指导，完成“散乱污”企业综合整治，化工企业入园率突破50%，减少非电行业煤炭消费完成省定任务，严格执行主城区禁放烟花爆竹规定，实现PM2.5浓度下降和空气质量优良天数比率上升。大力实施水系连通、河湖清淤、黑臭水体治理等重点工程，大幅消减入河（湖）污染物总量，全面消除国省考断面劣Ⅴ类水体，完成乡镇供水水源地达标建设。

加强生态保护修复。深化落实“三长一体”管护机制，开展领导干部自然资源资产离任审计，推进山水林田湖草系统生态治理，加大河湖“三乱”整治力度，压减洪泽湖养殖面积1.5万亩，新建生态样本河道40条，努力打造水清、流畅、岸绿、景美的河湖生态环境。实施化肥农药使用减量增效行动，试点轮作休耕面积2万亩，净增成片造林2.5万亩。全面推行农村公益设施“五位一体”管护模式，加快镇村污水管网建设，稳步提升建制镇污水集中处理率。

创新生态建设机制。深化生态环境保护体制机制改革，改进环保监管执法模式，完善失信联合惩戒机制，放大污染防治攻坚监督平台作用，系统化提升治理成效。严格执行项目建设环境准入负面清单与生态补偿、责任追究制度，从严开展“双随机”检查与各类专项整治行动，加强污染源监管执法网格化建设，严厉打击违法行为。推进省级以上开发区循环化改造，严格控制能源消费总量和强度，加快化工钢铁煤电行业转型升级高质量发展，积极创建国家和省级公共机构节能示范单位，坚决遏制能耗过快增长，力争淮阴、涟水生物质发电项目并网运行，淮安经济技术开发区争创国家级绿色园区。

（六）坚持建管并重，增强中心城市引领力。突出完善功能、提升品质、强化辐射，加快建设有地域特色、有文化特质、有时代特征的现代化区域中心城市。

着力打造城市特色。加快“多规合一”平台应用，注重系统规划设计和刚性管制，进一步厚植人文底蕴，放大资源优势，不断丰富提升生态文旅水城的城市内涵。坚持以水为脉，统筹实施水岸及码头整治、水文化复兴，提升古淮河、盐河及环洪泽湖、白马湖等廊道景观风貌，恢复清安河生态功能。突出以文为魂，深入挖掘运河文化内涵，加强清口水利枢纽、清江大闸、漕运总督公署、洪泽湖大堤等运河遗址遗产区保护，做好周恩来故里景区空间景观设计，加快建设板闸遗址公园、河下古镇、山阳湖、九龙口等历史文化风貌区，打造特色鲜明的大运河文化带标志城市。

加快完善城市功能。计划投入813亿元，实施600个重点项目。着力打造功能片区，推进淮海广场商业区、水渡口商务区业态升级，开工建设金融中心二期及风情街，增强核心商圈辐射力。推进高教园区、智慧谷等科技文创区建设，加速高端资源要素集聚。加快基础设施建设，完善综合客运枢纽功能，确保徐宿淮盐铁路顺利通车，建成连淮扬镇铁路连淮段，建设宁淮铁路与连淮扬镇铁路并行段，全面启动京沪高速公路扩建，推进348、420省道等干线公路建设和330省道前期工作，完成市区内环高架一期主体工程，建成淮海南路大运河桥。加快智慧淮安大数据管理服务平台建设。不断优化人居环境，加快城区已拆地块建设进度，改造棚户区4875户，新建一批省级宜居示范居住区。实施市区内环高架、翔宇大道沿线景观提升工程，优化街头游园等公共空间。健全公共设施，建设城市慢行系统、立体停车系统，增加公共停车泊位，增设20公里公交专用道，智能化改造交通信号设施，启动选址新建北京路水厂、四季青污水处理厂，加快建设焚烧发电厂、建筑垃圾处理厂，实施餐厨废弃物处理厂二期扩容，积极推进城市地下空间开发。

加强城市精细管理。进一步厘清管理职责权限，推动管理重心下移，实现精细化管理全过程、全覆盖。完成环卫作业市场化改革，全面加强城区公共场所环境卫生整治，扎实推进市区居民小区垃圾分类。不断完善全国文明城市创建长效机制，全面落实《淮安市文明行为促进条例》和《淮安市市容管理条例》，进一步加大市容环境整治力度，着力解决环境脏乱差、户外广告设置不规范、流动摊点占道经营等突出问题。强化城市管理综合执法，深入开展不文明行为专项整治，让讲文明守礼仪树新风成为市民的自觉。

（七）聚焦民富民享，持续提高群众获得感。立足人民群众对美好生活的新期盼，着力打通服务民生、联系群众最后一公里。

完善保障体系。全面落实创业扶持政策，安排4000万元引导资金和1000万元担保资金，重点扶持创业、再就业和脱贫项目。实施参保精准扩面，引导新业态从业人员参保，帮扶低收入困难群体参保。完善统一的城乡居民基本医保制度和大病保险制度，提升城乡居民养老保险一体化水平。加强大病保险与基本医保、医疗救助衔接，推进医保支付“三医联动”改革。充分保障残疾人、留守儿童等弱势群体利益，建成10个以上残疾人辅助性就业基地。开展养老机构服务质量提升行动，动员各类社会力量为居家老人提供上门服务，试点建设嵌入式养老机构，推动医养结合深入发展。继续加大老年教育工作保障力度。

优化公共服务。深化教育体制机制改革，创新教职工编制动态管理机制，推动公办幼儿园独立办学，落实义务教育学校管理标准，加快高品质示范高中建设步伐，促进民办学校分类管理，支持大中专院校融合发展。实施85个教育建设项目，改善30所学校教学装备水平，巩固提升校外培训机构专项整治成果。建立现代医院管理制度，构建紧密型医联体，加强全科医生队伍建设，更大力度推进分级诊疗，提升家庭医生签约服务质效，争创国家级临床重点专科、省级医学重点学科，加快建设苏北区域医疗高地。强化基层医疗卫生机构监督管理，确保公共卫生服务安全，打造一批国家和省级示范基层医疗卫生机构。组建市文旅集团、演艺集团，建立健全公共文化服务网络，加强文物保护利用和非物质文化遗产保护传承。新建体育公园37个、健身步道100公里以上，继续办好品牌体育赛事，促进群众体育、竞技体育和体育产业协调发展。

加强社会治理。推进新一轮平安淮安创建，加快“升级版技防城”建设，深入开展扫黑除恶专项斗争。提升社区网格化治理质效，推进社会矛盾多发、信访问题突出的重点领域专项治理，严防大规模群体性事件和重大公共安全事件。健全公共法律服务体系，拓展法律援助范围，做好信访、调解、仲裁、行政复议和社区矫正等工作，将矛盾妥善化解在基层。深入开展安全隐患排查整治执法行动，增强应急处置能力，严防重特大生产安全事故。启动国家食品安全示范城市创建，推进“透明食安”扩点连线成面。构建新时代国防动员体系，规范基层武装建设，加强退役军人服务管理保障，扎实做好救助慰问工作，推动军地融合协调发展，争创全国双拥模范城五连冠。

着眼解决好群众最期盼最迫切的民生问题，实施提标改造老旧小区环境、治理城市黑臭水体、保障高层住宅小区饮用水安全、提高城乡群众生活保障水平、让更多孩子入好园上好学、开展妇儿关爱行动、方便城乡群众公共出行、提升小餐饮店环境、帮助残疾人就业脱贫、丰富城乡群众文化体育生活十件民生实事项目，全面加强跟踪审计，确保将实事办实办好！

三、全面加强政府自身建设

新时代是奋斗的时代，我们是奔跑的追梦人。面对高质量跨越发展的新任务和人民群众追求美好生活的新期待，我们将坚持把党对一切工作的领导贯彻到政府工作各领域，更加自觉地把发展重任扛在肩上，把人民利益记在心上，努力打造人民满意的政府。

践行新理念，建设创新政府。坚持以系统性思维创造性贯彻新理念新战略，在统筹推进“五位一体”总体布局中，抓重点补短板强弱项，努力探索符合淮安实际的发展之路。坚持以开放性视野跳出淮安看淮安，自觉在全省全国的大局中找准定位、树好标杆，在对接实施国家和省重大战略上抢抓机遇、拓展空间、打造特色。坚持以现代化手段提升治理能力，全面推进观念更新、制度创新、模式出新。

强化新担当，建设效能政府。坚持用民意的“标尺”来丈量作风的“短板”，努力用自己的辛勤指数换取群众的幸福指数。始终发扬“钉钉子”精神，坚决打通政策执行最后一公里，务求工作干一件成一件。认真落实“三项机制”，坚持严管与厚爱结合、激励与约束并重，让广大干部在严格约束下奋发有为。深入贯彻集中整治形式主义、官僚主义的部署要求，扎实开展政府系统“中梗阻”专项治理，着力解决“上推下卸”等不作为、慢作为、乱作为问题，大力营造想干事、能干事、干成事的浓厚氛围。

适应新形势，建设法治政府。加强地方立法研究，深化司法体制改革，推进依法行政，严格规范公正文明执法。推动政务公开标准化规范化，覆盖权力运行和政务服务全过程，让政策传递更加顺畅、政府履职更加透明。加强合法性审查和风险评估，健全政府科学决策、民主决策、依法决策机制。认真做好行政复议和应诉工作，切实维护公平正义、保障群众权益。依法接受人大法律和工作监督、政协民主监督，主动接受司法监督、社会监督和舆论监督，确保行政权力在阳光下运行。

落实新要求，建设廉洁政府。自觉把全面从严治党要求贯彻到政府工作各个方面，切实增强“四个意识”，坚决做到“两个维护”，更大力度推进党风廉政建设。严格落实中央八项规定精神和实施细则要求，充分发挥领导干部“头雁效应”，有效防止“四风”变异。深化政府系统惩治和预防腐败体系建设，积极支持纪检监察机关监督执纪问责，深入查处各种不正之风和发生在群众身边的腐败行为，突出重点领域、关键岗位的审计监督和监察监督，促进干部清正、政府清廉、政治清明。

各位代表，风正时济，定当扬帆破浪；重任在肩，更需策马加鞭。我们将紧密团结在以习近平同志为核心的党中央周围，在省委省政府和市委的正确领导下，不忘初心再出发，牢记使命谱新篇，以优异的成绩庆祝新中国成立70周年，为推动淮安高质量跨越发展、高水平全面建成小康社会而努力奋斗！
